# Neonatal Joubert Syndrome With Renal Involvement and Respiratory Distress

**DOI:** 10.7759/cureus.24907

**Published:** 2022-05-11

**Authors:** Beena D Agarwal, Satya Mohapatra, Sumedha Singh, Vijay Guduru, Soumya R Nayak

**Affiliations:** 1 Radiology, Institute of Medical Sciences and SUM Hospital, Bhubaneswar, IND; 2 Radiology, Instiute of Medical Sciences and SUM Hospital, Bhubaneswar, IND

**Keywords:** molar tooth sign, respiratory distress, newborn, renal involvement, joubert syndrome

## Abstract

Joubert syndrome (JS) is a rare autosomal recessive neurodevelopmental disorder with characteristic clinical presentation of hyperpnea-apnea spells, hypotonia, dysmorphic facies, and nystagmus and imaging features of molar tooth sign and cerebellar vermian hypoplasia-dysplasia. Early diagnosis is needed for timely management and favorable outcome. We present a case of neonatal JS with renal involvement presenting with respiratory distress and highlight the characteristic clinical and imaging findings. On examination, the baby had low set ears, a large protruding tongue, hypertelorism, and a depressed nasal bridge. Ultrasonography (USG) abdomen showed echogenic kidneys with cortical and medullary cysts. Magnetic Resonance Imaging (MRI) brain showed classical molar tooth sign, vermian hypoplasia-dysplasia, and thinning of the corpus callosum.

## Introduction

Joubert syndrome (JS) is a rare autosomal recessive inherited disorder first described by French neurologist Marie Joubert in 1969. It has a reported incidence of 1:80,000 to 1:100,000 [1,2]. It presents with respiratory dysregulation, hypotonia, ataxia, developmental delay, and oculomotor findings like nystagmus. Characteristic imaging findings are molar tooth sign and vermian hypoplasia. It is called “classical JS” when it involves only the central nervous system (CNS) and “JS and related disorders (JSRD)” when it is associated with other organ involvement. JSRD includes JS with renal, ocular, oculorenal, hepatic, or orofaciodigital defects [3]. JS is considered a disease of variable phenotype with different ages of presentation, the average age of diagnosis being 33 months [4]. We hereby present a case of neonatal JS with renal involvement presenting with respiratory distress and highlight the characteristic imaging findings emphasising the value of a prompt diagnosis and multidisciplinary approach in the management of these patients.

## Case presentation

**Figure 1:**
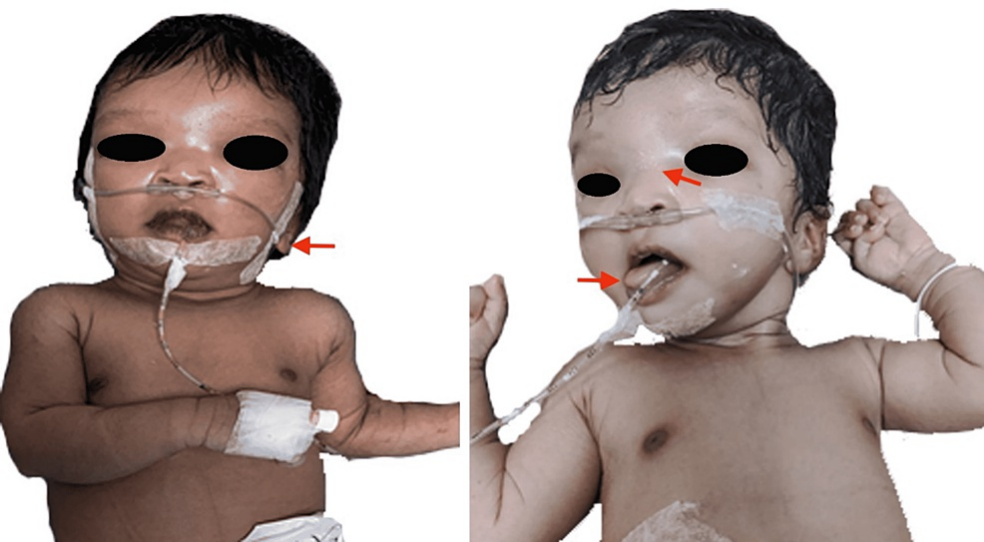
Neonate with low-set ears, depressed nasal bridge, hypertelorism, and a large protruding tongue.

A term female neonate delivered by vaginal delivery to a primigravid mother presented with signs of respiratory distress six hours after birth. The baby had cried immediately after birth. Examination revealed hypertelorism, low-set ears, depressed nasal bridge, large protruding tongue, hypotonia, and poor reflexes with an Appearance, Pulse, Grimace, Activity, and Respiration (APGAR) score of 7 (Figure 1). Vital signs were normal. Sepsis evaluation, arterial blood gas (ABG) analysis, and chest radiograph were normal. However, she had a few episodes of hyperpnea with no desaturation until day 4 of life, when the baby developed apnea with desaturation, she was kept on non-invasive ventilation (NIV) support. She had intermittent hyperpnea followed by apnea with spontaneous recovery after being weaned off from respiratory support. She also had intermittent desaturations for which she was kept on oxygen support. On day 10, the baby had a generalized tonic seizure which was managed with phenobarbitone. The course was uneventful thereafter and the baby was discharged on day 13.

**Figure 2:**
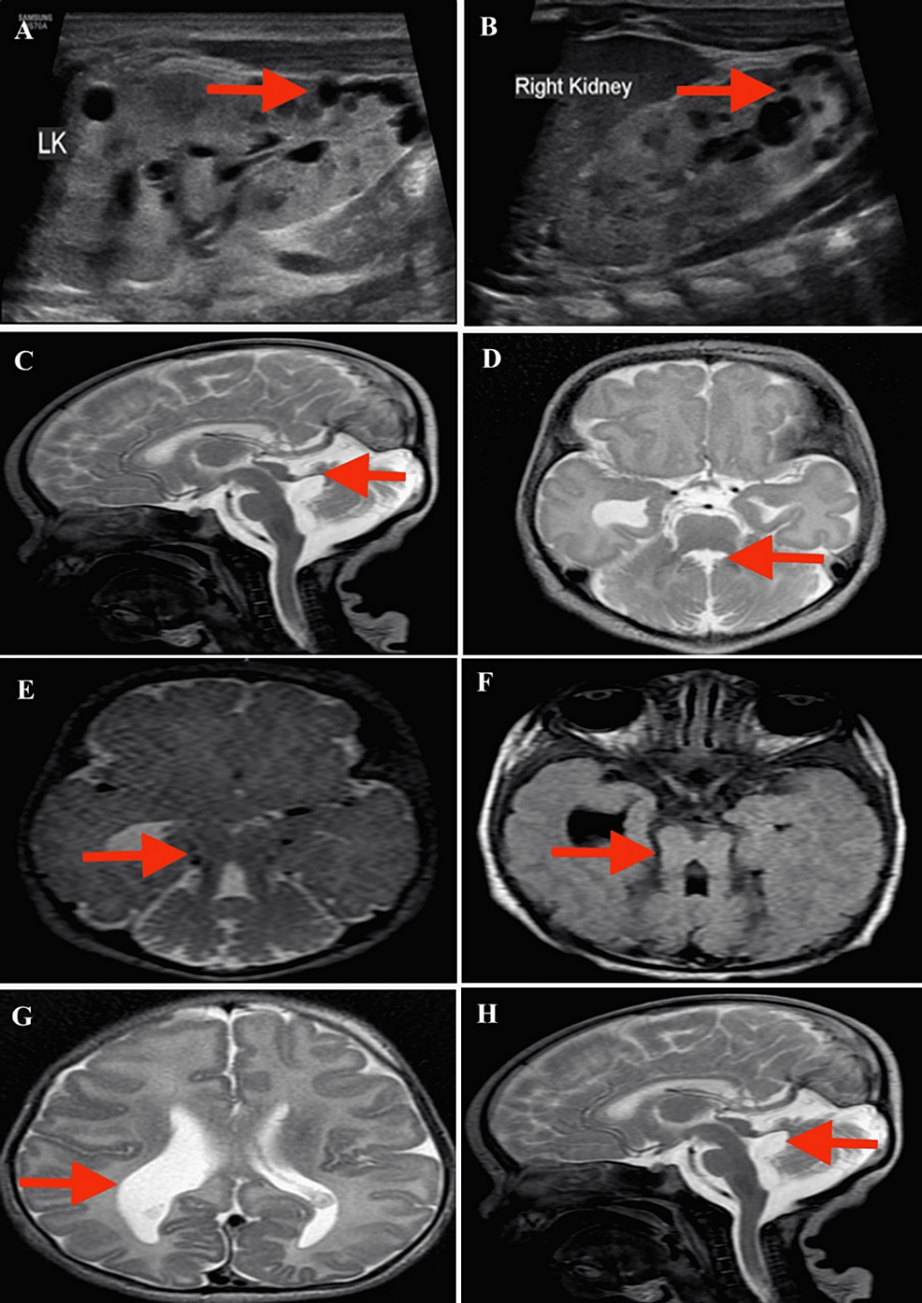
(A,B) USG of bilateral kidneys showed increased cortical echogenicity with reduced CMD and multiple small, simple cortical and medullary cysts. (C) Sagittal T2 MRI showed hypoplasia of the vermis and enlargement of the fourth ventricle with upward and posterior displacement of the fastigium. (D) Axial T2 MRI showed batwing appearance of the fourth ventricle. (E,F) Axial T2 and FLAIR MRI showed thick and elongated superior cerebellar peduncles with a deep interpeduncular fossa. (G) Axial T2 MRI showed asymmetrical dilatation of the right lateral ventricle. (H) Sagittal T2 MRI showed thinning of the corpus callosum. USG, ultrasonography; CMD, corticomedullary differentiation; MRI, Magnetic Resonance Imaging; FLAIR, fluid-attenuated inversion recovery

Given the dysmorphic facial features, screening neurosonography (NSG), ultrasonography (USG) abdomen, and Magnetic Resonance Imaging (MRI) brain were advised. NSG was done with the HS70 Samsung USG machine (Samsung, Seoul, Korea) with both curvilinear and linear array transducer probes and showed asymmetric dilatation of the right lateral ventricle and enlarged posterior fossa. USG abdomen showed normal-sized bilateral kidneys with increased cortical echogenicity, reduced-to-lost corticomedullary differentiation (CMD), and a few simple sub-centimetric cortical and medullary cysts. The liver was normal in size with a contracted gall bladder. The portal vein, common bile duct, and intrahepatic biliary radicles were normal. The rest of the abdominal organs were unremarkable (Figure 2A, 2B).

MRI brain was performed on GE 1.5T MRI machine (GE Healthcare, Chicago, USA) using a head coil and mild sedation. Axial T1 and T2, fluid-attenuated inversion recovery (FLAIR), susceptibility-weighted imaging (SWI), coronal and sagittal T2, coronal FLAIR, and diffusion-weighted imaging (DWI) sequences were obtained. They showed normal myelination for age, thickened and elongated bilateral superior cerebellar peduncles with deep interpeduncular fossa giving the appearance of a molar tooth, severe vermian hypoplasia causing upward and posterior displacement of fastigium with resultant dilatation of the fourth ventricle giving a batwing appearance, thinning of the corpus callosum, and asymmetric dilatation of right lateral ventricle (Figure 2C-2H). Ocular examination and 2D-Echocardiography were normal. 

## Discussion

Joubert syndrome is termed a ciliopathy as it involves mutations in genes whose products are located in and around the primary cilium which plays an important role in signaling pathways of multiple organs, predominantly the brain, kidney, and retina. Our case report adds to the medical literature on the syndrome by highlighting clinical and radiological features of the same, emphasising the value of a prompt diagnosis and early management. To date, mutations in 34 genes have been recognized, out of which 33 are inherited in an autosomal recessive pattern and one in an X-linked recessive pattern [5,6]. The term JSRD is reserved for patients who present with classical radiological and clinical features of JS along with other organ involvement. JS is classified into six types - pure JS, JS with ocular defect (JS-O), JS with renal involvement (JS-R), JS with oculorenal defects (JS-OR), JS with hepatic defect (JS-H), and JS with orofaciodigital defects (JS-OFD) [1]. Common ocular defects include retinal dystrophy, renal defects include nephronophthisis (medullary cystic disease complex), hepatic defects include congenital hepatic fibrosis, and orofaciodigital defects include lobulated tongue and multiple oral frenula, mesoaxial polydactyly with Y-shaped metacarpals, and cleft lip/palate [7]. This syndrome can also be broadly classified into two groups based on associated retinal dystrophy. Patients with retinal dystrophy have associated medullary cystic kidney disease and a poorer survival rate than those without retinal dystrophy [8].

The clinical presentation is characterized by dysmorphic facial features, abnormal breathing patterns, abnormal ocular movements, hypotonia, developmental delay, and ataxia. Its classical clinical triad includes infantile hypotonia, developmental delay, and abnormal eye movements or respiratory dysregulation [9]. Dysmorphic facial features include low set ears, hypertelorism, large protruded tongue, prominent forehead, and depressed nasal bridge [10,11]. Respiratory dysregulation is classically seen in the neonatal period and the incidence diminishes with age [12,13]. It is characterized by hyperpnea which worsens with stimulation, followed by a period of apnea or episodic hyperpnea alone [14]. 

Oculomotor apraxia is the most common ocular feature characterized by the inability to track smooth pursuits and loss of vestibulo-ocular reflex [3,11]. Renal involvement occurs in 25-33% of patients. Autosomal recessive polycystic kidney disease, multicystic dysplastic kidney disease, and nephronophthisis are the most common forms. USG is characterized by increased cortical echogenicity with diminished CMD and cortical and medullary cysts [15]. 

Joubert syndrome is diagnosed on imaging by the classical MRI findings of molar tooth sign (thickened and elongated superior cerebellar peduncles and deep interpeduncular fossa), batwing or umbrella sign (cerebellar vermian hypoplasia-dysplasia resulting in the abnormal configuration of the fourth ventricle), and dysplastic pontomesencephalic junction with abnormal decussation of superior cerebellar peduncles. Other supratentorial findings include corpus callosal dysgenesis and lateral ventricular enlargement [3].

Besides JS/JSRD, molar tooth sign is associated with other syndromes like COACH, Varadi-Papp, Dekaban-Arima, Senior-Loken, and Malta. However, these syndromes have prominent supplementary features to suggest an alternative diagnosis [4]. Cerebellar vermian hypoplasia is also seen in conditions like Dandy-Walker malformation (DWM) and rhombencephalosynapsis. However, communication of the fourth ventricle with an enlarged posterior fossa in DWM and fusion of both cerebellar hemispheres in rhombencephalosynapsis suggest respective diagnoses [5]. Detailed clinical evaluation of the patient for the classical clinical triad and imaging features can suggest the diagnosis of JS and exclude other causes. Once the diagnosis of JS/JSRD is suggested, a detailed ocular assessment with electroretinogram (ERG) and slit-lamp examination, and renal and hepatic assessment with USG and urine challenge test should be done [4]. The diagnosis of JS is also important for future procedures that require anesthesia since these patients are sensitive to respiratory depressant effects of anesthetic agents like opiates and nitrous oxide [16].

## Conclusions

JS is a rare inherited neurodevelopmental disorder having variable symptoms and characteristic imaging features. Combining the clinical symptoms of developmental delay, hypotonia, respiratory dysregulation, and abnormal ocular movements, and the imaging features of molar tooth sign and vermian cerebellar hypoplasia, a diagnosis of JS can be suggested. Further evaluation with necessary investigations should be done to look for other organ involvement. Early screening and diagnosis can predict the patient’s clinical outcome and impact the management. 
